# Updates on the Virulence Factors Produced by Multidrug-Resistant *Enterobacterales* and Strategies to Control Their Infections

**DOI:** 10.3390/microorganisms11081901

**Published:** 2023-07-27

**Authors:** Mohd W. Azam, Raffaele Zarrilli, Asad U. Khan

**Affiliations:** 1 Medical Microbiology and Molecular Biology Laboratory, Interdisciplinary Biotechnology Unit, Aligarh Muslim University, Aligarh 202002, India; 2 Department of Public Health, University of Naples Federico II, 80131 Naples, Italy

**Keywords:** virulence, multidrug-resistance, *Enterobacterales*

## Abstract

The *Enterobacterales* order is a massive group of Gram-negative bacteria comprised of pathogenic and nonpathogenic members, including beneficial commensal gut microbiota. The pathogenic members produce several pathogenic or virulence factors that enhance their pathogenic properties and increase the severity of the infection. The members of *Enterobacterales* can also develop resistance against the common antimicrobial agents, a phenomenon called antimicrobial resistance (AMR). Many pathogenic *Enterobacterales* members are known to possess antimicrobial resistance. This review discusses the virulence factors, pathogenicity, and infections caused by multidrug-resistant *Enterobacterales*, especially *E. coli* and some other bacterial species sharing similarities with the *Enterobacterales* members. We also discuss both conventional and modern approaches used to combat the infections caused by them. Understanding the virulence factors produced by the pathogenic bacteria will help develop novel strategies and methods to treat infections caused by them.

## 1. Introduction

Bacteria cause thousands of infections and pathogenesis in both plants and animals, including human beings. The severity of pathogenicity varies from one bacterial species to another bacterial species. Virulence factors are the factors responsible for the pathogenicity of the organism. Many specific virulence factors play roles in the disease process. Pathogenic *E. coli*, a representative member of *Enterobacterales*, can produce both structural (such as capsular polysaccharides, flagella curli, fimbriae, and pili) and secreted (such as iron-acquisition systems and toxins) virulence factors that contribute to the pathogenicity [1,2]. Gram-positive (GPB) and Gram-negative bacteria (GNB) cause several infections in different organisms. Accordingly, bacterial infections can be grouped into Gram-positive bacterial infections and Gram-negative bacterial infections.

The family Enterobacteriaceae of order *Enterobacterales* contains over 53 genera and 238 species, and about 95% of the clinically most important species belong to 10 genera and less than 25 species. Some *Enterobacterales* members are common inhabitants of the small and large gastrointestinal tracts (GI), and therefore they are sometimes called enterics. Most of the members are facultative anaerobes, oxidase negative, and glucose fermenters, and reduce nitrates to nitrites. All members have peritrichous flagella with few exceptions; *Klebsiella* and *Shigella* are non-motile [3,4,5,6,7]. *Enterobacterales* are an extensively distributed heterogeneous group of bacteria. They contribute about 80% of Gram-negative isolates, causing many diseases in human beings. The members of *Enterobacterales* are the causative agents of causing urinary tract infections (UTIs), hospital- and healthcare-associated pneumonia, diarrhea, meningitis, bloodstream infections, sepsis, endotoxic shock, various intra-abdominal infections, and many other infections. The most common species of this order to cause human infections are *Escherichia*, *Klebsiella*, *Salmonella, Yersinia*, *Proteus*, *Enterobacter*, *Shigella*, *Citrobacter,* and many others [8,9]. The most common diseases caused by *Enterobacterales*: *Klebsiella* spp. and *Enterobacter* spp. cause pneumonia, *Escherichia coli* is a common cause of UTIs, and *Salmonella* causes gastroenteritis. In addition to this, almost all *Enterobacterales* members are involved in bloodstream infections (BSIs) and intra-abdominal infections like cholangitis, peritonitis, and others. An increase in the antibiotic resistance in *Enterobacterales*, especially extended-spectrum beta-lactamases (ESBLs) and carbapenemase-producing *Enterobacterales* (CRE), creates a major problem for treating the infections caused by these bacteria [8,10].

*E. coli* is a member of *Enterobacterales*, and it is the most abundant facultative anaerobe in the gastrointestinal (GI) tract of a human being. They live as nonpathogenic strains in the GI tract, and many strains cause diseases in normal and immunocompromised persons [3]. *E. coli* is a much-diversified group of organisms and is divided into three groups: probiotic or commensal strains, intestinal–pathogenic strains (InPEC or Diarrheagenic *E. coli*), and extraintestinal–pathogenic strains (ExPEC) [11]. Based on diseases caused by *E. coli,* they are grouped into different pathotypes. These pathotypes include intestinal pathogenic *E. coli* strains (e.g., Shiga toxin-producing *E. coli*) and extraintestinal pathogenic *E. coli* strains (e.g., UPEC) [12]. 

Commensal *E. coli* represents ecologically important, nonpathogenic inhabitants of intestinal tracts of humans and other animals. These commensal *E. coli* can change into pathogenic strains from nonpathogenic strains and vice versa. Both pathogenic and nonpathogenic *E. coli* strains can colonize the gut and are very well adapted to the large intestine environment [13]. 

Probiotics are live microorganisms that are beneficial to health when taken in adequate amounts. The common attributes related to probiotics are that they can colonize the human intestine; resist gastric, bile, and pancreatic secretions; and attach to epithelial cells. The common probiotic microorganisms in use are *E. coli* Nissle 1917, lactic acid bacteria *Lactobacillus*, *Bifidobacterium,* and the yeast *Saccharomyces boulardii* strain. Probiotics can be administered as pharmaceutical products and as dietary supplements for therapeutic purposes [14]. 

*E. coli* Nissle 1917 (EcN) is a well-known probiotic strain. It is known to play a positive role in gastrointestinal homeostasis and microbiota balance and has several therapeutic health benefits, including inducing and maintaining ulcerative colitis (UC) reduction. EcN also regulates the host immune response and modulates anti-inflammatory effects. In addition to this, EcN strengthens the intestinal epithelial barrier and increases the expression of antimicrobial factors such as β-defensin-2 and microcins [15,16].

Diarrheagenic *E. coli* (intestinal–pathogenic *E. coli* InPEC) cause gastroenteritis or colitis in humans. Six different diarrheagenic *E. coli* (DEC) pathotypes have been well described [11]. The intestinal pathogenic strains of *E. coli* causing human infections are Exterotoxigenic *E. coli* (ETEC), causing diarrhea in children; Enterohemorrhagic *E. coli* (EHEC), producing cytotoxins called Shiga toxins and leading to bloody diarrhea, hemolytic uremic syndrome (HUS), hemorrhagic colitis, and severe conditions that may lead to death; enteroinvasive *E. coli* (EIEC), causing watery diarrhea; enteroaggregative *E. coli* (EAEC), causing persistent diarrhea in both children and HIV-infected patients; enteropathogenic *E. coli* (EPEC), causing infantile diarrhea and vomiting; and diffusely adherent *E. coli* (DAEC), causing diarrhea in young children [3,4]. Sepsis-causing *E. coli* (SEPEC), uropathogenic *E. coli* (UPEC), and neonatal meningitis-associated *E. coli* (NMEC) are placed under extraintestinal pathogenic *E. coli* (ExPEC) [17]. ExPEC is a common cause of urinary tract infections (UTIs), neonatal meningitis, bone and joint infections, and nosocomial pneumonia [3,4]. 

Urinary tract infections (UTIs) are one of the most frequent infections of bacteria in human beings. UTIs are caused by bacteria (both Gram-negative and Gram-positive) and yeast (e.g., *Candida* spp.). Uropathogenic *E. coli* (UPEC) is the leading cause of community-acquired UTIs, and it contributes about 90% of all UTIs globally. UTIs primarily affect sexually active premenopausal women and create a burden for both the economy and health sectors [18]. They affect 50% of all women at least once in their lifetime. After respiratory infections, UTIs are the second most common human infections, which result in an annual economic burden of about USD 3.5 billion [19]. Based on their genetic similarity, *E. coli* strains are further sub-classified into eight phylogenetic clades or phylogroups: A, B1, B2, D, E, F, and G [12,18]. The increase in antibiotic resistance and the emergence of multidrug-resistant strains (*E. coli* ST131) have created a need to find alternative methods to treat these infections [19].

### 1.1. Virulence Factors Produced by E. coli and Other Enterobacterales Members

**Figure 1:**
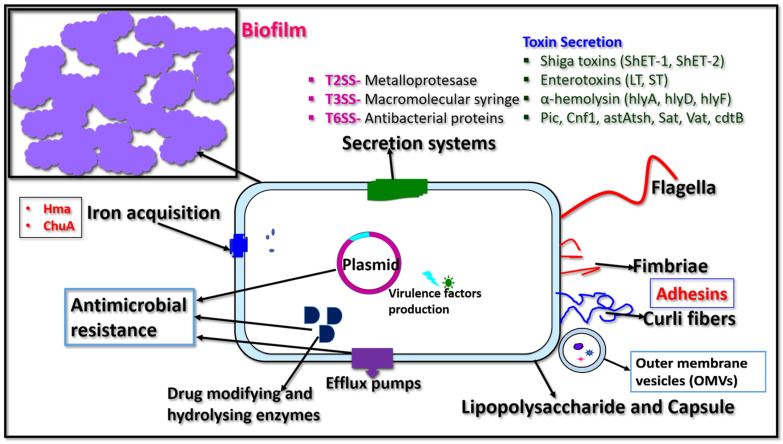
Showing the cartoon image of virulence factors produced by the bacteria belonging to *Entorobacterales*.

A wide range of virulence factors are produced by *E. coli* and other members of *Enterobacterales* that contribute to their pathogenicity (Table 1; Figure 1). The most common virulence factors are discussed below.

#### 1.1.1. Adhesins

Adhesins are a group of proteins involved in the attachment and colonization of pathogenic bacteria to biotic (e.g., in human intestines) and abiotic surfaces (e.g., steel or plastic). Many bacterial species produce several adhesins with specific receptor-binding properties. Most adhesins behave as lectins, which recognize the oligosaccharide moieties of glycolipids or glycoproteins [66,67]. *Enterobacterales* pathogenic members produce a wide range of adhesins, including fimbriae/pili, curli, and outer membrane proteins. Cell surface carbohydrates sometimes also play the role of adhesins. Type 1 fimbriae are the most common adhesins among *Enterobacterales* pathogens [68]. Type 1 fimbriae (Fim), N-acetyl d-glucosamine-specific fimbriae (Gaf), P fimbriae (Pap/Prf), temperature-sensitive hemagglutinin (Tsh) N-acetyl d-glucosamine-specific fimbriae (Gaf), M-agglutinin (Bma), S/F1C fimbriae (Sfa/Foc), afimbrial adhesin (Afa), and bifunctional enterobactin receptor/adhesin (Iha) are some common adhesins produced by pathogenic *E. coli* [17].

#### 1.1.2. Fimbriae

The terms “fimbriae” and “pili” are often used interchangeably. However, the term “pilus” should be used for cellular appendages involved in genetic material transfer, i.e., conjugation, and “fimbriae” should be used for structures that have a role in bacterial adhesion to various surfaces. On average, bacterial cell surfaces contain more than 400 fimbriae and 1–10 conjugative pili. Fimbriae are rod-shaped structures 5–10 nm diameter and are involved in cellular adhesion. Conjugative pili are similar structures to fimbriae except that they are longer than fimbriae. Pili are primarily made up of pilin proteins and are organized into a tube-like system through which genetic material passes during conjugation [69,70]. Type 1 fimbriae are the most prevalent fimbriae and are found in more than 80% of pathogenic *E. coli* and other pathogenic members of ***Enterobacterales***. The expression of type 1 fimbriae is regulated by pathogenicity islands (PAIs), and they play a significant role in UPEC virulence, especially in the case of urinary tract infections. The FimH adhesin protein of type I fimbriae specifically binds to α-D-mannose residues bound to membrane glycoproteins present on enterocyte cell surfaces, bladder cells, and brain capillary endothelial cells. Other important fimbriae, S fimbriae, are involved in adhesion to intestinal and urinary tract cells; F1C fimbriae are involved in bladder and kidney endothelial cell adhesion [11]. 

#### 1.1.3. Curli and Amyloid Fibers

Curli fibers are the extracellular proteinaceous fibers produced by several *Enterobacterales* members, including *Escherichia coli* and *Salmonella* spp. They are the first functional amyloid identified in bacteria and contribute to the major proteinaceous part of the extracellular matrix in *E. coli* biofilms. In *E. coli* and *Salmonella* spp., they play an essential role in several physiological and pathogenic processes. They help with cell aggregation, surface colonization, surface adhesion, and biofilm formation. Curli fibers also mediate cell adhesion and host cell invasion, interact with host factors and the host immune system, and are potent inducers of inflammatory response. The curli biogenesis and structure are unique among the bacterial fibers known till now. Curli fibers are 4 to 7 nm thick, insoluble in SDS (sodium dodecyl sulphate), and have a characteristic cross beta-strand structure that gives structural integrity to the curli fibrils. Biophysical and biochemical studies have revealed that curli belong to a class of fibers called amyloids. Amyloid fiber formation (amyloidogenesis) is a causative agent of several human diseases, such as Parkinson’s, Huntington’s, prion, and Alzheimer’s diseases. However, functional amyloids in bacteria are produced by highly regulated and coordinated biosynthetic processes [30,71,72].

### 1.2. Secretion System

The protein secretion systems or secretion systems are essential to bacteria for their growth and are involved in several viral processes. Pathogenic bacteria have developed a number of methods to enter hosts, damage tissues, and skip the host immune response. As a part of these strategies, many pathogenic bacteria secrete proteins across the phospholipid membranes. The secreted proteins perform many roles to increase bacterial virulence. They enhance bacterial attachment to eukaryotic cells, intoxicate target cells, disrupt their normal cell machinery, and so on. Many pathogenic bacterial cells have exclusive protein secretion systems for the secretion of virulence factors from bacterial cytosol into the host cell environment [73].

Several types of secretion systems exist in bacteria. Among them, T2SS, T3SS, T4SS, T5SS, T6SS, and T7SS are the most common types. The type II secretion system (T2SS) secretes various enzymes and toxins from periplasmic space to the external environment. For example, in EHEC, O157:H7 plasmid-encoded T2SS secretes metalloprotease, which degrades the C1 esterase inhibitor, which plays a role in complement activation. Similarly, another T2SS encoded on chromosomes secretes chitinase [68]. 

The pathotypes of diarrhea-causing bacteria *Salmonella* spp., *E. coli*, *Yersinia* spp., and *Shigella* spp. use the type III secretion system (T3SS) to infect the host. T3SS acts as a macromolecular syringe, and Gram-negative bacteria use it to deliver effector proteins (virulence factors) to the target cells or host cells. The T3SS extends across the outer and inner bacterial membranes and penetrates into the plasma membrane of the host cell like a syringe to inject or deliver the effector proteins from bacteria to the host cytosol. This structure allows the bacteria to control the signaling pathways of the host cell and create an ecological niche to survive. In some *E. coli* pathotypes, the T3SS translocates more than 25 effector proteins [74]. The type VI secretion system (T6SS) is associated with bacterial virulence and plays several roles in pathogenic bacterial strains. T6SS kills neighboring non-immune bacteria after cell–cell contact by secreting antibacterial proteins directly into the periplasm of the target bacteria. It helps with host intestine colonization under conditions of high bacterial competition for resources. Some T6SSs are directly associated with pathogenesis, such as macrophage survival and biofilm formation. The pervasiveness of T6SS in pathogenic *E. coli* strains suggests its essential role in virulence. Bacterial T6SS is also associated with virulence to eukaryotic host cells, but few T6SSs are directly involved in cell disruption. Pathogens with T6SS constitute a significant threat to human health, like *Escherichia coli, Salmonella typhimurium*, *Yersinia pestis*, *V. cholerae,* and many others [37].

Outer membrane vesicles (OMVs) are proteoliposomal spherical vesicles that originate from the outer membrane of Gram-negative bacteria. These nanosized (20–250 nm) vesicles are known to play important roles during host–microbe interactions, biofilm formation, and pathogenesis [75,76]. Many Gram-negative bacterial species, especially *Enterobacterales* members, are known to produce OMVs, such as *E. coli* [*Enterotoxigenic E. coli (ETEC), Shiga toxin-producing E. coli (STEC)*, *Enterohemorrhagic E. coli (EHEC)*], *Salmonella typhi*, *Yersinia pestis*, *Shigella flexneri*, *Cronobacter* spp., etc. [77]. OMVs possess a diversity of endogenous cargos that have several biological roles. These OMV cargos are mature macromolecules that mediate the transfer of a number of biological molecules. They include extracellular proteins, DNA fragments, cytotoxins, virulence factors, autolysins, and many others. Their production enhances the relationship between the bacteria and the host and aids with inter- and intra-species communication. OMVs are involved in a variety of physiological and pathological functions. To date, it has been discovered that OMVs play a part in pathogenicity, acquisition of nutrients, stress responses, biofilm formation, delivery of toxins, parental bacterial protection, bacterial community communication, adhesion, and virulence factors to evade the host defense system [76,77,78]. Some of the best examples of OMVS include the *E. coli* Ail protein, *IpaB, IpaC,* and *IpaD* of *S. flexneri* [77].

### 1.3. Toxins

Toxins are among the best-studied virulence factors in bacteria, and bacteria secrete them into the outside environment or target host cells. During the infection, toxins play a crucial role, as they help with the bacterial invasion to host tissues, increasing cytotoxicity and unresponsiveness to neutrophils. Enterohaemorrhagic *E. coli* (EHEC) with the *stx* gene for Shiga toxin (Stxs) production is generally referred to as Shiga toxin-producing *E. coli* (STEC) or as verocytotoxin-producing *E. coli* (VTEC). STEC strains cause hemorrhagic colitis (HC). *Shigella dysenteriae* also possesses the *stx* gene, which produces endotoxins (ShET-1 and ShET-2), rendering hemolytic uremia and colitis [21,68]. ETEC strains secrete two types of enterotoxins (heat-labile enterotoxin (LT) and a heat-stable enterotoxin (ST)) that cause diarrhea or traveler’s diarrhea [79]. ExPEc strains produce a number of toxins, like α-hemolysin (hlyA, hlyD, hlyF), a protease involved in colonization (pic), cytotoxic necrotizing factor 1 (cnf1), enteroaggregative *E. coli* toxin (astA), temperature-sensitive hemagglutinin tsh autotransporter (tsh), secreted autotransported toxin (sat), vacuolating autotransporter protein (vat), and cytolethal distending factor (cdtB) [21].

### 1.4. Lipopolysaccharides and Capsules

Lipopolysaccharides (LPS) are the major component of the Gram-negative bacterial outer membrane. They play a crucial role in host–pathogen interactions and the innate immune system. *Enterobacterales* LPS is an extremely potent virulence factor, and it is mainly due to signaling through the TLR4 pathway. Lipid A is a biologically active component of LPS and an endotoxin, and it is recognized by the pattern recognition receptors, e.g., Toll-like receptor 4 (TLR4), of the host. A highly variable O antigen of LPS is also crucial for pathogenesis and immunity to *Enterobacterales* infections [68,80].

### 1.5. Iron Acquisition

Iron (Fe) is the essential element for *E. coli* survival. Iron is involved in many cellular activities, like electron transport chain (ETC/ETS), nucleotide biosynthesis, and peroxide reduction. *E. coli* cells (e.g., ExPEC including uropathogenic *E. coli*) have evolved many methods to acquire iron from the host or site of infection. The acquisition may be a direct or indirect method. *E. coli* cells can acquire iron directly from the free form of heme or proteins with heme, like hemoglobin or hemopexin. Some heme-specific receptors, like Hma and ChuA, bind to hemoproteins and transfer them to the periplasm. From the periplasm, an ABC transport system further transports them to the cytoplasm. The indirect method involves a shuttle system that utilizes siderophores as ferric iron chelators [81]. 

### 1.6. Antimicrobial Resistance

A continuous rise in antibiotic resistance (AR) is a significant threat for health care clinicians treating the infections caused by resistant bacteria in both developing and developed nations. The intensive use and especially the misuse of antibiotics is the main reason behind the emergence of antibiotic resistance (AR). The emergence of antibiotic resistance (AR) or antimicrobial-resistant (AMR) strains in Gram-negative bacteria, including *Enterobacterales* and *E. coli,* has made treatment of infections caused by them very difficult [82,83]. AMR bacteria, particularly *K. pneumoniae* and other *Enterobacterales* that produce ESBL and carbapenem resistance (CR), are able to develop resistance very quickly against the various antimicrobial agents, creating a limitation in available options for treatment of infections, including UTIs, pneumonia, and sepsis, leading to increased morbidity [2,84]. The emergence of resistance to polymyxin has also been reported in *Enterobacterales* [82,83,85,86]. The acquisition of resistance or virulence factors gives the microorganism an advantage for survival [87]. A bacterial cell acquires resistance genes by horizontal gene transfer (HGT) and also by mutations. In one of the earlier studies, we have showed bla_NDM-1_ and its variants to be in association with other markers like bla_OXA-48_, bla _CTX-M_ and bla _KPC-2_ [82,83,88]. Mutations are spontaneous, and their frequency depends on the type of microorganism and antimicrobial agent. Horizontal gene transfer has a very important role in the spread of antimicrobial resistance genes and bacterial evolution. In HGT, the bacterial cells acquire foreign DNA by three mechanisms: conjugation, transduction, or transformation. The lateral transfer or HGT of resistance genes occurs by mobile elements such as transposons, plasmids, or integrons. Intrinsic resistance is also found in *E. coli* and possesses genes conferring resistance against certain antibiotics like beta-lactamase, aminoglycosides, and fluoroquinolones (Table 2) [83,89]. An inverse correlation has been described between high colistin resistance and virulence of *K. pneumoniae* clinical blood isolates [82,83,86].

### 1.7. Plasmids

Plasmids are extrachromosomal genetic material. *Escherichia coli* possess many types of plasmids, including the plasmids associated with virulence. All *E. coli* pathotypes, ETEC, EIEC, EPEC, EHEC, EAEC, and ExPEC, have plasmids essential for their virulence. Although many types of plasmids exist in *E. coli* and almost all virulence plasmids, they belong to a single category known as IncF [90]. Virulence plasmids generally occur in low copy numbers, are large (>40 kb), and encode host–pathogen interaction-promoting genes. Many strains of EHEC O157:H7 serotype possess virulence plasmid pO157, which helps with the adherence of bacterial cells to epithelial cells of the intestine and leads to hemolytic uremic syndrome (HUS). In EPEC, pB171 plasmid (~69 kb) can be found, and it contributes to adherence to epithelial cells of the intestine. EIEC possess pINV plasmid, and virulence genes encoded on it are closely related to those on *Shigella* spp. ETEC has a typical pCoo plasmid that encodes for a range of virulence factors, including toxins and colonization factors that vary from strain to strain. EAEC possess pAA plasmid, and they encode for a diverse range of toxins and fimbriae. *Yersinia enterocolitica* and *Yersinia pseudotuberculosis* are the leading cause of foodborne and zoonotic yersiniosis and possess pYV plasmid crucial for their virulence. The pINV plasmid in *Shigella* spp. is essential for virulence and helps the bacterium to invade epithelia [91].

### 1.8. Flagella

Bacterial flagella and motility play several roles in virulence and bacterial pathogenesis. Flagella serve several crucial functions on surfaces or hydrogels, including cell adhesion, biofilm formation, and host–pathogen (bacteria) interactions. Flagellar motility increases the colonization of the host at the early stages of infection and can work as adhesins. During the initial phase of the disease, motility helps move through the mucus layer and initial contact setup with the host epithelium. *E. coli* possess peritrichous flagella. The variable antigenic domain of FliC shows different seroreactivity. On the basis of seroreactivity, *E. coli* flagella are divided into H-serotypes. More than one pathotype may contain similar H serotypes, and different H-serotypes may be found in strains belonging to one pathotype. In the case of the STEC strain of the EHEC pathotype, the flagella were involved in invasion and were not directly involved in bacterial adhesion [92,93]. 

### 1.9. Biofilm

Microbial biofilms are a surface-attached, structured community of microorganisms enclosed in a matrix they secrete themselves. Biofilm matrix mainly contains polysaccharides, proteins, nucleic acid, water, and ions. The bacterial cells in biofilms can tolerate hostile environmental conditions such as desiccation, starvation, or lack of nutrients, which enable them to cause a wide range of chronic infections. Biofilm defends the bacterial cells against the host immune system through impaired activation of the complement system and phagocytes. In addition, it increases bacterial resistance by about a thousandfold against the commonly available antibiotics [94]. Biofilm formation increases the survival of the bacterial population and enhances the pathogenic ability of the microorganism. Horizontal gene transfer is also associated with biofilm formation [95,96]. Bacterial cells in the biofilm have a different lifestyle than planktonic cells. Biofilm promotes antibiotic resistance and DNA exchange in bacteria. Biofilms are associated with chronic and persistent human infections, and the genesis of about 65% of hospital-acquired infections is from biofilm. *E. coli* biofilm-like intracellular bacterial communities (IBCs) and biofilms of neonatal nasogastric feeding tubes have a crucial role in UPEC pathogenesis because the formation of IBCs enables UPEC to continue bladder colonization and resist removal. The ExPEC cells are able to exchange DNA in biofilms, which is a matter of concern because of the possibility of acquisition of plasmids with antimicrobial resistance and other virulence factors [97]. Bacterial biofilm persistence is a leading source of recurrent or chronic infections in the human body [2,98].

## 2. Strategies to Combat Bacterial Infections

Several strategies are in use to combat bacterial infections (Table 3). These strategies include both traditional and novel methods (alternative methods).

### 2.1. Conventional or Traditional Approaches

#### 2.1.1. Antibacterial Agents

In this strategy, various antimicrobial agents/antibiotics with different targets (e.g., cell wall, ribosomal subunits, DNA replication, folic acid metabolism, etc.) are often used as anti-infective agents. Such antibiotics belong to different chemical groups, have different action mechanisms, and show different activity against Gram-positive and Gram-negative bacteria.

The most common group of antibiotics include:

- Beta-lactamases—Beta-lactams target penicillin-binding proteins (PBPs) primarily and make them unavailable for new peptidoglycan synthesis. This peptidoglycan synthesis disruption causes bacterial cell lysis.
- Cephalosporins—hydrolyze the ester and amide bond of beta-lactam rings.
- Fidaxomicin (macrocyclic antibiotics)—It inhibits RNA polymerase and prevents transcription.
- Glycopeptides—target the D-alanyl D-alanine peptide side chain of the peptidoglycan precursor subunit. Vancomycin inhibits cell wall synthesis by blocking the D-alanyl subunit binding with the PBP.
- Quinolones—inhibit the bacterial DNA gyrase enzyme.
- Aminoglycosides—bind with the 16S rRNA 30S subunit and cause misreading and premature termination of mRNA translation.
- Pleuromutilins—retapamulin, a pleuromutilin derivative, binds to domain V of 23S rRNA and inhibits protein synthesis in bacteria.
- Macrolides—inhibit protein synthesis by targeting peptidyl transferase, leading to premature peptide chain detachment.
- Oxazolidinones—interfere with protein synthesis by binding to the 23Sr RNA of the 50S subunit and interacting with peptidyl-t-RNA [99,100,101,102].

#### 2.1.2. Antibiofilm Agents

Antibiofilm agents are a diverse group of compounds that may be obtained through synthetic or natural sources. Synthetic compounds include metal chelating agents, lantibiotics, nanoparticles, analogs, and derivatives. Several nanoparticles like silver (AgNPs) and gold (AuNPs) are effective antibiofilm agents and can kill bacteria in established biofilms produced by several clinical strains. Many antibiofilm agents are derived from several organic compounds such as imidazole, phenols, and indoles [101,103]. A wide range of naturally occurring compounds shows antibacterial activity or antibiofilm activity. Emodin is a natural anthraquinone extracted from the barks and roots of many plants, lichens, and molds, and is reported to inhibit biofilm formation. Phloretin, a flavonoid, was reported to control biofilm formation in *E. coli* O157:H7 by inhibiting fimbriae production. Ginger extracts have been observed to reduce *P. aeruginosa* biofilm formation. Some natural compounds with antibiofilm activities are chelerythrine, isolimonic acid, ginkgolic acid, carvacrol, and casbane diterpene [103]. 

#### 2.1.3. Inhibition of Quorum Sensing

Quorum sensing (QS) is used to regulate the gene expression in bacteria depending upon their cell density, which acts as a critical factor in regulating the production of virulent factors and causing infection [104]. Most bacterial infectious diseases are caused by biofilm and mediated by quorum sensing (QS). Several bioactive compounds from prokaryotes and eukaryotes have been identified to disrupt biofilms. These molecules mainly act by quenching, and this phenomenon is also called quorum quenching (QQ). Synthetic molecules have also been found to be useful as QS inhibitors. The QS system can be disturbed or inhibited by several methods: by synthesizing the chemical analogs of signal molecules, inhibition of QS signals by antibody and decoy receptors, degradation of QS signal molecules through enzymes, inhibition of QS signal molecules synthesis, degradation of N-acyl homoserine lactones (AHLs), or AHL synthase or AHL cognate receptor protein activity reduction [105,106].

#### 2.1.4. Disruption of Bacterial Amyloids

It has been reported that analogs of FN075 and BibC6 of ring-fused 2-pyridones act as inhibitors of the *E. coli* curli biogenesis. The urinary tract infection mouse model shows a significant reduction in virulence upon treatment with FN075. This compound has blocked the biogenesis of both type 1 pili and curli, which have a major role in *E. coli* biofilm formation. In *Bacillus subtilis* biofilms, TasA protein forms functional amyloid-like fibers. Parthenolide (a sesquiterpene lactone) and AA-861 (a benzoquinone derivative) inhibit amyloid-like fibers in *Bacillus subtilis* biofilms. AA-861 inhibited the TasA protein, forming functional amyloid-like fibers [104].

#### 2.1.5. Dissolution of eDNA

Extracellular DNA (eDNA) is a crucial constituent of the extracellular polymeric substance of microbial biofilm. eDNA plays an essential role in bacterial cell adhesion and aggregation, resulting in stabilizing and maintaining biofilm integrity. In addition to this, several other functions, such as cation chelators and nutrient sources, are also attributed to eDNA. In *P. aeruginosa*, QS signals control the release of eDNA in biofilms. Due to its role in biofilm and cell aggregation, several antibiofilm approaches targeting eDNA have been proposed. For example, recombinant human DNase I treatment of staphylococcal biofilms prevents biofilm formation and leads to the detachment of biofilms. DNase I treatment of *P. aeruginosa* resulted in the dissolving of mature biofilms. In another study, antibiofilm activity against *P. aeruginosa* biofilms was reported, in which ciprofloxacin-loaded poly(lactic-co-glycolic acid) nanoparticles were activated with DNase I [107,108].

#### 2.1.6. DNA Topoisomerase Inhibitors

DNA topoisomerases are exploited as one of the therapeutic targets for antibacterial agents. Quinolones (e.g., fluoroquinolones) are the most common antibacterial drugs targeting bacterial type IIA topoisomerases. The development of fluoroquinolone resistance in pathogenic bacteria created an urgent need to search for novel antibacterial agents. Some recent inhibitors against topoisomerase have been found to be effective against fluoroquinolone-resistant pathogens. They include the inhibitors against type IIA topoisomerase, which may form nick-containing ternary complexes or interact with the GyrB/ParE subunit. Besides type IIA topoisomerase inhibitors, some topoisomerase I inhibitors have recently been identified [109].

### 2.2. Alternative Approaches to Combating Bacterial Infections

The rise in bacterial antimicrobial resistance (AMR) reduces the available drug options to treat the infections caused by them and increases the need to find alternative strategies to treat the diseases.

#### 2.2.1. Photodynamic Therapy (PDT)

PDT uses non-toxic photosensitizers (PS), which, upon excitation at a specific wavelength by harmless visible light, produce cytotoxic reactive oxygen species (ROS) and free radicals. Photodynamic therapy (PDT) provides an alternative with an efficient approach to fight against biofilm-related microbial infections. Many PDT studies performed both in vitro and in vivo showed a remarkable biofilm reduction or eradication. PDT is helpful in combating several clinically significant biofilms, like ventilator-associated pneumonia, dental biofilms, chronic wound infections, chronic rhinosinusitis, and oral candidiasis. ROS produced by activated photosensitizers (PS) target and damage biomolecules such as nucleic acids, lipids, and proteins present on the cell surface, inside the cells, or in the biofilm matrix. This nonspecific damage of biomolecules leads to the eradication of both planktonic cells and biofilms [110,111]. 

#### 2.2.2. Antioxidants to Control Biofilm

Several genes related to biofilm formation and quorum sensing (QS), as well as other virulence factors, have been found to be inhibited by antioxidants in *M. smegmatis*, *P. aeruginosa*, *Campylobacter*, *Streptococcus*, and *B. subtilis*. Antioxidants are compounds with free radical scavenging or neutralizing properties. They are less cytotoxic than antibiotics or other chemicals; therefore, they can be used as an alternative source to control biofilm-associated infections. Antioxidants’ ability to react with free radicals is primarily related to conjugated double-bond structures. This reaction may occur with oxygen moieties (e.g., curcumin) or in the absence of oxygen (e.g., carotenes). In addition, antioxidants react in the presence of functional groups such as phenolic and polyphenolic groups (e.g., gallic acid, catechin, and tannic acid). Plants are an abundant source of antioxidants. Several studies have been performed to explore the antibiofilm potential of antioxidant-rich plant extracts to inhibit biofilms and various biofilm-forming pathogens [107]. Baicalin was extracted from the roots of *Scutellaria baicalensis*. It has antimicrobial, antioxidant, anticancer, and anti-inflammatory properties and can be used to treat a variety of diseases [112].

#### 2.2.3. Smart Materials against Bacteria

Zwitterionic polymers such as poly(sulfobetaine) (PSB), poly(phosphataine) (PPB), and poly(carboxybetaine) (PCB) contain a negatively charged group (e.g., phosphate, carboxylate, or sulfate phosphate) and a positively charged quaternary ammonium salt (QAS). Due to their distinct hydrophilic and anti-fouling properties, zwitterionic polymers are widely used in biomedical engineering. Switchable temperature-responsive surfaces are other examples of smart materials used as bactericidal and anti-fouling surfaces. These surfaces facilitate the release of bacteria by switching from relatively hydrophilic at lower temperatures and facilitating bacterial adherence by switching to relatively hydrophobic at higher temperatures. Poly(N-vinylcaprolactam) (PVCL) and poly(N-isopropylacrylamide) (PNIPAAm) have been used as temperature-responsive polymers [113]. In a recent study, silver nanoparticles (AgNPs) of various shapes were encapsulated within a hydrogel matrix of polyacrylamide (PAA), and N-methylenebisacrylamide (MBA) (PAA-MBA) showed shape-dependent antimicrobial and mechanical properties [114].

#### 2.2.4. Enzymatic Degradation of Biofilms

The degradation of the extracellular matrix of biofilms by some enzyme proteases, DNases, and glycosidases (e.g., DNase I, a-amylase dispersin B) leads to the dispersal of biofilm cells and the release of planktonic cells and their components. It makes planktonic cells easier to be targeted by antibiotics. Since proteins constitute a considerable part of the biofilm matrix, proteases can be considered one of the biofilm eradication enzymes with the most potential for use. Proteases from staphylococcal strains such as staphopain A and B, aureolysin, spl protease, and proteinase K can degrade biofilms. The LapG protein of *Pseudomonas putida* alters the exopolysaccharide-binding protein LapA, leading to biofilm degradation [115,116].

#### 2.2.5. CRISPR-Cas in Infection Control

CRISPR-Cas is now a well-established molecular biology tool for genome editing, with a vast range of applications in a large number of genomes of different organisms. Due to their high specificity, CRISPR-Cas systems can be used to target the pathogenic bacterial genome and detect infectious diseases caused by bacteria, viruses, and parasites. Marraffini and colleagues proposed that CRISPR/Cas9 systems can be applied to kill pathogenic bacteria in a sequence-specific manner. CRISPR can also be used to immunize bacteria against the spread of multidrug-resistant (MDR) plasmids. CRISPR has been repurposed for different applications. “Smart antibiotics” have been developed by CRISPR, repurposed to avoid multidrug resistance and distinguish between beneficial and harmful microorganisms [117,118,119]. In addition, the CRISPR-Cas system can be utilized to re-sensitize antibiotic-resistant bacteria by targeting and eradicating the antibiotic resistance genes harboring plasmids [119]. Researchers have used the subtype I-E CRISPR-Cas system for selective killing of *Salmonella enterica* and *E. coli* strains in pure and mixed culture experiments. Phagemid-mediated delivery of RNA-guided Cas9 was used in enterohemorrhagic *E. coli* (EHEC) to delete the *eae* gene encoding a virulence factor for bacterial adhesion to epithelial cells of the host. Beta-lactamase genes NDM-1 and SHV-18 of *E. coli* were also successfully targeted and removed [120]. Cas9 protein has been found to be a potential therapeutic agent, and it has been developed as an antimicrobial agent that can be applied to target antimicrobial-resistant or virulent bacterial strains [121].

## 3. Conclusions

Virulence factors produced by bacteria play different roles for bacteria, including an increase in survival. *Enterobacterales* members produce many pathogenic factors, which is the leading cause of their increased pathogenic potential and infections. The involvement of antimicrobial-resistant species in these infections further increases the complications of diseases and makes these infections very difficult to treat. Therefore, there is a dire need to gain a better understanding of the virulence and pathogenic factors of *Enterobacterales* and other bacteria in order to develop novel methods and strategies to treat the infections caused by them.

## Figures and Tables

**Table 1:** Virulence factors produced by *Enterobacterales*.

Virulence Factor Category	Virulence Factor	Role(s) in Virulence	Genes	Organisms	Refrences
**Adhesin**	Type 1 Fimbriae	Adhesion, colonisation, invasion	*fim* gene family *fim* A-I	*E. coli*	[20,21]
	Type 3 Fimbriae	Adhesion, colonisation	*mrkABCDF* operon	*E. coli*, *Klebsiella pneumoniae*	[22,23]
	F1C Fimbriae	Adhesion, colonisation	*focA*, *focC*, *focD*, *focf*, *focG*, *focH*, *focI*	*E. coli*	[24]
	S Fimbriae	Adhesion, colonisation	*sfaA-H*, *sfaS*, *sfaX*, *sfaY*	*E. coli*	[25]
	P Fimbriae	Adhesion, colonisation	*papA-K*	*E. coli*, *P. mirabilis*	[26]
	Auf Fimbriae	Adhesion, colonisation	*aufA-G*	*E. coli*	[25]
	F9 Fimbriae	Adhesion, colonisation	*c1931-c1936*	*E. coli*	[25]
	Stf fimbriae	Adhesion, colonisation	*stf*	*Salmonella* spp.	[27]
	Saf fimbriae	Adhesion, colonisation	*saf*	*Salmonella* spp.	[27]
	Dr	Adhesion, colonisation	*draA-draE*, *draP*	*E. coli*	[28]
	Afa	Adhesion, colonisation	*afaI-afaIV*, *nfaI*, *drII*	*E. coli*	[29]
	Curli amyloid fibers	Components of the biofilm extracellular matrix, surface colonisation	*csgBA* and *csgDEFG* operons	*E. coli*, *Salmonella* spp.	[30]
**Autotransporter protein**	Temperature-sensitive hemagglutinin	Autotransporter protein	*tsh*	*E. coli*	[31]
	SPATE (serine protease autotransporters of *Enterobactericeae*)	Diverse function s (adhesin, protease, esterase, lipase, etc.)	*sat*, *pic*	*E. coli*, *Shigella*, *Salmonella*	[32]
**Secretion system**	T1SS	Toxin secretion	T1SS operon	*E. coli*	[33]
	T3SS	Injects effector proteins into the host cells	T3SS genes	*E. coli* EPEC, *E. coli* EHEC, *Salmonella* spp., *Shigella*	[34]
	T5SS	Autotransporters	T5SS genes	*E. coli*	[35]
	T6SS	Secretion of antibacterial proteins and many others	*sci-1* and *sci-2 clusters*	*E. coli*	[36,37]
**Toxins**	Lipid A	Endotoxin		*Salmonella* spp., *E. coli*	[38]
	Alpha-hemolysin (HlyA)	Cytotoxic agent	*hlyCABD operon*	*E. coli, Staphylococcus aureus*	[39]
	Colibactin (Clb)	Genotoxic molecule	*clb* gene cluster	*E. coli*	[40]
	Colicin	Antimicrobial proteins	Colicin operons	*E. coli*, *Enterobacter cloacae*	[41]
	Cytolethal distending toxin (CDT-I to CDT-V)	Cytotoxic agent, block eukaryotic cell cycle	*cdt* operon	*E. coli*, *Shigella dysenteriae*, *Salmonella enterica*, *Campylobacter* spp., *Aggregatibacter actinomycetemcomitans*, *Escherichia albertii*, *Haemophilus ducreyi*, *Helicobacter* spp., *Providencia alcalifaciens*,	[42]
	Cytotoxic necrotizing factor 1 (CNF-1)	Inflammation and tissue damage	*cnf1*	*E. coli UPEC*	[43]
	Hemolysin (HlyF)	Induces autophagy in eukaryotic cells	*hlyF*	*E. coli*	[44]
**Lipopolysaccharide and capsule**	Capsular polysaccharides (K antigen)	Adherence, resistance to host immune system	K-antigen cluster	*E. coli*, *Acinetobacter baumannii*, *Burkholderia pseudomallei*, *Vibrio* spp.	[45,46,47]
	O-antigen	Adherence and help to overcome host defense mechanisms	O-antigen cluster	*E. coli*, *Salmonella enterica*	[48]
**Iron acquisition**	Siderophores (Enterobactin, Bacillibactin)	Iron acquisition	Siderophore biosynthesis genes	*E. coli*, *K. pneumoniae*	[49]
**Antimicrobial resistance**	Beta-lactamase	Resistance to beta-lactam antibiotics	*bla*_TEM_, *bla*_CTX–M_, *ampC*	*E. coli*, *Klebsiella* spp.	[50,51]
	Aminoglycoside resistance	Resistance to aminoglycoside antibiotics	Genes encoding aminoglycoside-modifying enzymes (AGMEs)	*E. coli*, *Klebsiella* spp.	[52]
	Fluoroquinolones	Resistance to fluoroquinolone antibiotics	*qnr* genes	*E. coli*, *Klebsiella* spp., and other *Entorobacterales members*	[53]
**Plasmids**	pO157	Helps with the adherence of bacterial cells to epithelial cells of the intestine	*ehxA*, *etpC to etpO*, *espp.*, *katP*, *toxB*, *ecf*, *stcE* etc.	EHEC O157:H7	[54]
	pB171	Contribute to adherence to epithelial cells of the intestine	*parABC locus*, etc.	EPEC	[55,56]
	pINV	Essential for invasiveness	ipa–mxi–spa locus etc.	*E. coli* EIEC, *Shigella* spp.	[57,58]
	pCoo	Toxins and colonisation factors	*cooB*, *A*, *C*, *D*, etc.	ETEC	[59]
	pAA	Toxins and fimbriae	*Pet*, *pic*, *senB*, *orf3, orf4*, *aar*, *capU*, *virK*, *shf*, etc.	EAEC	[60]
	pYV	Encodes a type III secretion system required for plasmid-borne anti-host factors delivery called Yops	*ysc*, *lcr*, yop, etc.	*Yersinia enterocolitica* and *Yersinia pseudotuberculosis*	[61]
**Flagella**	**Flagella**	Early biofilm formation, adherence, and invasion	*flhDC operon*	*E. coli*, *S. enterica*, *Salmonella* and other *Entorobacterales members*	[62,63]
**Biofilm**	**Biofilm**	Increases the survival of the bacterial population and enhances the pathogenic ability of the microorganism	*fimAICDFGH* operon quorum sensing genes, e.g., *lusS* and *pfs*, etc.	*E. coli*, *S. enterica*, *Salmonella*, and other *Entorobacterales* members	[64,65]

**Table 2:** Three types of antimicrobial or antibiotic resistance (AMR) in bacteria: intrinsic, acquired, and adaptive.

Intrinsic	Acquired	Adaptive
Innate ability to resist antimicrobial agent through its inherent structural or functional characteristics. Independent of environmental stimuli Efflux pumps Production of antibiotic-inactivating enzymes (AmpC) Decreased outer membrane permeability Regulators (ArcA) Can be transmitted vertically to subsequent generations, e.g., *Enterobacter* spp., *Citrobacter* spp.	Acquired through the acquisition of resistance genes through horizontal gene transfer and chromosomal gene mutations. Independent of environmental stimuli Efflux pumps upregulation Diminished permeability Production of antibiotic-inactivating enzymes (endogenous beta-lactamases expression, acquired beta-lactamases, aminoglycoside-modifying enzymes, ESBL, 16S rRNA, methylases, carbapenemases) Target site mutations DNA gyrase and topoisomerase IV mutations LPS modification Regulators Can be transmitted vertically to subsequent generations, e.g., *K. pneumoniae*, *E. coli*	Inducible resistance occurring due to the presence of antimicrobial agents and (or) other environmental stresses. Environmental stimuli dependent (e.g., pH, heat shock, DNA stress, polyamines, biocides, oxygen, subinhibitory levels of antibiotics, growth state, media, etc.) Alterations in gene and/or protein expression Regulators Biofilm formation Swarming motility Twitching motility Transient existence and normally reverts once the causing condition is removed, e.g., *K. pneumoniae, E. coli*

**Table 3:** Strategies to combat bacterial infections. These strategies include both conventional and alternative approaches.

Conventional Approaches	Alternative Approaches
Antibacterial agents Antibiofilm agents Inhibition of quorum sensing Disruption of bacterial amyloids Dissolution of eDNA DNA topoisomerases inhibitors	Photodynamic therapy (PDT) Antioxidants to control biofilm Smart materials against bacteria Enzymatic degradation of biofilms CRISPR-Cas system

## Data Availability

No new data were created or analyzed in this study. Data sharing is not applicable to this article.
